# Microbial Infection and Rheumatoid Arthritis

**DOI:** 10.4172/2155-9899.1000174

**Published:** 2013-12

**Authors:** Song Li, Yangsheng Yu, Yinshi Yue, Zhixin Zhang, Kaihong Su

**Affiliations:** 1 Department of Pathology and Microbiology, University of Nebraska Medical Center, Omaha, NE 68198, USA; 2 The Eppley Institute for Research in Cancer and Allied Diseases, University of Nebraska Medical Center, Omaha, NE 68198, USA; 3 Department of Internal Medicine, University of Nebraska Medical Center, Omaha, NE 68198, USA

**Keywords:** Rheumatoid arthritis, Infection, Microbes, Etiopathogenesis

## Abstract

Rheumatoid arthritis (RA) is a complex autoimmune disease affecting 1–2% of general worldwide population. The etiopathogenesis of RA involves the interplay of multiple genetic risk factors and environmental triggers. Microbial infections are believed to play an important role in the initiation and perpetuation of RA. Recent clinical studies have shown the association of microbial infections with RA. Accumulated studies using animal models have also found that microbial infections can induce and/or exaggerate the symptoms of experimental arthritis. In this review, we have identified the most common microbial infections associated with RA in the literature and summarized the current evidence supporting their pathogenic role in RA. We also discussed the potential mechanisms whereby infection may promote the development of RA, such as generation of neo-autoantigens, induction of loss of tolerance by molecular mimicry, and bystander activation of the immune system.

## Introduction

Rheumatoid arthritis (RA) is one of the most common inflammatory autoimmune diseases. It is characterized by persistent synovitis, systemic inflammation and production of autoantibodies [1]. The molecular mechanisms of RA pathogenesis are not fully understood. It is believed that approximately half of the risk factors for RA are attributed to genetic factors such as the human leukocyte antigen (HLA) alleles while the other half of the risks are environmental factors including infection and smoking [2]. Clinical and animal model studies have suggested that infections by many microorganisms, such as *Porphyromonas gingivalis* (*P. gingivalis*), *Proteus mirabilis* (*P. mirabilis*), Epstein–Barr virus (EBV), and mycoplasma contribute to the etiopathogenesis of RA (Table 1).

For this review, we first identified the most common microbial infections associated to RA in the literature [3–5] and then performed a key word search using “arthritis” and “name of the microorganism” for original publications in English in the databases including Pubmed/Medline, Embase, EBSCO, SCOPUS, and Cochrane Library till November, 2013. The candidate microorganisms included in our search were *P. gingivalis, P. mirabilis*, EBV, cytomegalovirus (CMV), human immunodeficiency virus (HIV), parvovirus, hepatitis virus, herpes virus, human T-lymphotropic virus 1 (HTLV-1), mycoplasma, *Streptococcus pyogenes* (*S. pyogenes*), Salmonella, mycobacterium, and enterobacterium. Thus, this review will discuss studies regarding to those microorganisms with RA and emphasize on *P. gingivalis* which shows the strongest association with RA. Our discussion is organized in three sections, namely, clinical association of infection with RA, induction of arthritis by infection in animal models, and the pathogenic mechanisms of infection in RA.

## Clinical Association of Infection with RA

### Clinical co-existence of infection and RA

Periodontal disease (PD) is the most commonly associated RA disease. The association between the two has been considered since the early 1820s. PD is caused by chronic infection of approximately twenty different bacterial species, of which *P. gingivalis, Prevotella intermedia, Tannerella forsythia,* and *Aggregatibacter actinomycetemcomitans* are the most common ones. PD can progress from gingivitis to periodontitis and cause bone degeneration in the jaw. Clinical association studies consistently show that the prevalence of periodontitis is increased about two-fold in RA patients than non-RA patients. In a large study involving 4461 participants aged 60 or older in the US population, subjects with RA were more likely to have periodontitis (odds ratio (OR)=1.82) or complete tooth loss (edentulism, OR=2.27), compared to non-RA subjects after adjusting for age, gender, race/ethnicity, and smoking [6]. Another study reported that moderate to severe periodontitis was more prevalent in RA patients (51%) than age and gender matched osteoarthritis patients (26%) [7]. A recent study in the Dutch population confirmed the higher prevalence of severe periodontitis in RA patients [8]. They also reported that RA patients with severe periodontitis had higher DAS28 scores than RA patients with no or moderate periodontitis, suggesting that the severity of periodontitis is related to the severity of RA [8].

However, it is less clear that whether subjects with PD have increased incidence of RA. In a large prospective study involving 81,132 American women in the Nurses’ Health Study cohort, there is no increased risk of later-onset RA in subjects with a history of periodontal surgery and/ or tooth loss compared to subjects with healthy periodontal conditions [9]. In another large prospective study using the National Health and Nutrition Examination Survey cohort, subjects with PD experienced higher odds of prevalent/incident RA, but most odd ratios were not statistically significant [10]. It is also important to keep in mind that both studies are not specifically designed to examine the relationship between PD and RA. It is quite possible that there are missing data about PD and RA status in these cohorts and differential RA and PD ascertainment bias may also complicate the interpretation of data. Taken together, clinical studies have clearly shown the association of periodontal infection with RA. However, more longitudinal studies using well-defined populations are necessary to support the conclusion that periodontal infection is a risk factor for the development of RA.

Another common infection associated with RA is *Proteus*-caused urine tract infection. Patients with RA had significantly increased incidence of urinary tract infection and subclinical/asymptomatic bacteriuria compared to non-RA subjects [11]. *P. mirabilis* bacteria were isolated at a higher rate from urine samples of both female (63%) and male (50%) patients with RA than from healthy female (32–35%) and male (7–11%) subjects and patients with other autoimmune diseases including osteoarthritis, fibromyalgia, and psoriasis [12]. These studies implicate a plausible role of *Proteus* microorganisms in the development of RA.

Furthermore, it has been well documented that infections by a range of bacteria and viruses frequently manifest rheumatic diseases, including reactive arthritis. Gastrointestinal or genitourinary infections with *Salmonella, Shigella, Campylobacter, Yersinia*, and *Chlamydia trachomatis* may cause inflammatory oligoarticular or polyarticular sterile arthritis, usually starting within four weeks of infection [13]. Viruses including HIV, parvovirus, hepatitis viruses B and C, alpha-viruses like Chikungunya can cause acute or chronic forms of arthritis, and in some cases, mimic RA [13]. The precedence of infection over clinical arthritis suggests a causal relationship of the two events.

In summary, the clinical association studies suggest that infection is a risk factor for the development of RA. In addition, antibiotics such as sulphasalazine, minocycline, and rifampicin have been reported to be beneficial for the treatment of RA [14,15]. Reversely, periodontal treatments (oral hygiene and supragingival scaling) decreased the DAS28-CRP scores in RA patients [16], further implicating the pathogenic role of microbial infection in RA.

### Presence of microbial contents in RA tissues

Besides the disease association, the presence of microbial contents in RA tissues provides additional evidence for the correlation between infection and RA. Molecular techniques such as PCR and DNA/RNA-*in situ* hybridization have been widely used to detect bacterial or viral infections. *P. gingivalis* [17], mycoplasma [18,19], parvovirus [20], EBV [21,22], and cytomegalovirus (CMV) [22,23] have been identified in the synovial fluid, synovial membranes or serum samples from RA patients. Herpes viruses were detected in salivary cells and circulating lymphocytes from RA patients [24,25]. Besides nucleic acids, other microbial components can be used in the detection of infections in RA patients. Bacterial fatty acids, peptidoglycan, and muramic acid quantified by techniques such as gas-liquid chromatography (GLC), enzyme-linked immunosorbent assays (ELISA) and mass spectrometry have been used to detect the presence of microbes in RA samples [26–29]. For example, in a cohort of patients with early RA before any specific treatment, analyses of bacterial cellular fatty acids by GLC revealed the abundance of anaerobic bacteria in the intestinal flora of RA patients, implicating a possible role of intestinal anaerobic bacteria in the development of RA [26–29].

### Immune response to microbes in RA patients

Another strategy to detect previous and ongoing infections is to measure the immune responses to microbial components in patients. Indeed, antibodies against infectious microbes were detected in the sera of early RA patients and the levels of these antibodies correlated with the disease activity of RA. For example, elevated levels of IgM and IgA antibodies to *P. mirabilis* were found in rheumatoid factor (RF)-positive early RA patients [30]. The levels of anti-*P. mirabilis* antibodies in RA patients went down after one year of treatment and this decrease was significantly correlated with the decrease in a modified Stoke disease activity index in RA patients [31]. The specific antigens from P. mirabilis were later identified as haemolysin and urease [32,33].

Another prominent example is that increased antibody responses to *P. gingivalis*, one of the common bacteria causing PD, were detected in RA patient sera and synovial fluid. Furthermore, the anti-*P. gingivalis* antibody levels were correlated with the titers of anti-cyclic citrullinated peptide (CCP) antibodies (the recently added RA diagnosis criteria) in RA patients [34,35]. Interestingly, a recent study showed that anti-*P. gingivalis* antibodies were significantly associated with the presence of RA-related autoantibodies (anti-CCP and rheumatoid factor) in individuals at high risk of RA [36]. This result supports the hypothesis that infection by *P. gingivalis* may play a central role in the early loss of self-tolerance that occurs in the pathogenesis of RA. Increased antibody responses to other infectious agents, such as EBV [37], B19 parvovirus [38], and mycoplasma [39,40], have also been reported in RA patients. Furthermore, T cell responses to EBV [41–43] and CMV [44] were detected in the inflamed joints from RA patients.

In summary, clinical studies using human materials revealed a possible causative link between microbial infection and RA. However, more definitive studies in well characterized cohorts are necessary before we can conclude that microbial infection plays a crucial role in the initiation and perpetuation of RA. Moreover, the “chicken or egg” relationship between infection and RA may be hard to address in human studies. First, the RA-susceptible genetic and environmental factors, such as predisposed genes and living habits, may cause increased risks to infection even before or in the early stage of RA. Second, the RA-associated abnormal immune response and immunosuppressive medicine may contribute to decreased host defense to infection [45]. In this case, studies using animal models are very useful as experimental animals normally have homogeneous genetic and environmental backgrounds.

## Induction of Arthritis by Infections in Animal Models

Animal studies can directly address the causal relationship between infection and RA in accordant to Koch’s postulates, although there is no perfect animal model for human RA yet. Infections by *P. gingivalis* or mycoplasma induced or aggravated experimental arthritis in mice or rats [46–50]. Interestingly, a very recent study showed that *P. gingivalis* facilitated the development and progression of destructive arthritis in CIA mice through its unique bacterial peptidylarginine deiminase (PPAD) [51]. PPAD can lead to the generation of RA related citrullinated autoantigens by converting protein arginine residues to citrulline. This result suggests that *P. gingivalis* infection may play an important role in the loss of tolerance to citrullinated proteins in RA, implicating the causative link between infection and RA. In another study, experimental arthritis was strongly attenuated in the K/BxN mouse model under germ-free (GF) conditions, featured with reduced Th_17_ cells. Furthermore, introduction of segmented filamentous bacteria into GF mice reinstated the production of autoantibodies and arthritis symptoms [52]. This study shows that a single commensal microbe can drive the autoimmune arthritis possibly via its ability to promote Th_17_ cells.

Components derived from infectious pathogens can also induce or potentiate arthritis in animal models. For example, bacterial cell wall extracts can induce chronic arthritis in certain susceptible rat strains; and bacterial lipopolysaccharide (LPS) potentiates type II collagen-induced arthritis in mice [53,54]. Interestingly, EBV does not infect mice; and in a humanized mice model, EBV induced erosive arthritis with many features resembling those of RA [55,56]. Human T-lymphotropic virus 1 (HTLV-1) transgenic mice also developed inflammatory arthropathy, resembling RA [57].

In summary, the animal studies provide direct evidence for the causal relationship between infection and RA in experimental arthritis. Combined with the clinical association between infection and RA in human patients, it is convincing that microbial infection contributes to the etiopathogenesis of RA. However, the translation of findings from animals to humans is still arbitrary to some extent. First, adjuvant is used in the immunization protocol in these arthritis animal models. Adjuvant is known to strongly boost immune responses, and in the mean time, may also invoke immune responses to self-antigens. Second, the methods of delivering microbes in animal studies may differ from the routes of microbial evasion in humans. ird, arthritis symptoms in animals only resemble the joint inflammation in human RA, which may not represent the natural course of RA development in human patients. Nevertheless, these problems are common for animal of human diseases, and arthritis animal studies certainly provide valuable information for our understanding of RA pathogenesis.

## Pathogenic Mechanisms of Infection in RA

### Generation of neo-autoantigens

Autoantibodies play a crucial role in the development of RA. In fact, serum RF and anti-citrullinated protein antibodies (ACPAs) are included in the *2010 ACR/EULAR* diagnostic criteria of RA [58,59]. ACPAs are highly specific for RA and appear years earlier than the clinical diagnosis of RA [60]. Protein citrullination is a post-translational modification catalyzed by the enzyme peptidylarginine deiminase (PAD). *P. gingivalis* is the only prokaryotic organism that contains PAD. Recent studies showed that *P. gingivalis-*mediated citrullination of bacterial and host proteins provided an important mechanism for generating neo-antigens that drive the ACPA responses in RA [61]. Endogenous citrullinated proteins such as citrullinated α-enolase were abundant in *P. gingivalis*; and *P. gingivalis* PAD can citrullinate human proteins including common RA antigens fibrinogen and α-enolase [62]. Interestingly, a recent animal study showed that *P. gingivalis* facilitated the development and progression of destructive arthritis through its bacterial PAD enzymatic activities [51]. Microbial infections may also facilitate the citrullination process by activating host monocytes and neutrophils which express high levels of PAD [63–65].

Neutrophil extracellular trap (NET) is a structure released by activated neutrophils. It is composed of decondensed chromatins and granular molecules which can enhance the killing of extracellular microbes. It has been shown that bacterial LPS and some inflammatory cytokines can strongly induce NET. NET provides a source of autoantigens for several autoimmune diseases, such as vasculitis, systemic lupus erythematosus, and RA [66,67]. NET contains citrullinated RA autoantigens including α-enolase and vimentin. Furthermore, netting neutrophils have been found in synovial tissues, rheumatoid nodules, and skin from RA patients [67], implicating a role of NET in the pathogenesis of RA.

Collectively, microbial infections directly or indirectly induce the generation of citrullinated neo-autoantigens which may trigger the aberrant immune responses in RA [68].

### Loss of tolerance by molecular mimicry

Molecular mimicry plays an important role in the loss of tolerance in autoimmunity. Microbes may have elements that are similar in amino acid sequences or structure to self-proteins thus trigger autoantibody production through epitope spreading. For example, the *P. gingivalis* enolase and human α-enolase share 82% homology at the 17-amino acid immunodominant regions. Therefore, antibodies against bacteria enolase can recognize the homologous human α-enolase and promote the production of anti-human α-enolase autoantibodies. Indeed, the levels of anti-citrullinated human α-enolase antibodies were tightly correlated with the levels of antibodies to bacterial α-enolase in RA patients [69]. In addition, the affinity-purified antibodies to the human α-enolase peptide displayed cross-reactivity with the *P. gingivalis* enolase peptide [69]. Other examples include antibodies against EBV peptide p107 cross-react with the denatured human collagen and keratin [70]. Molecular mimicry also promotes autoreactive T cell activation and proliferation. The mimicry peptides for T cell activation are generally shorter and more linear compared to those of B cells. *E. coli* heat shock protein DnaJ contains a QKRAA motif that is also present in the HLA-DRB1 shared epitopes. DnaJ strongly activated RA synovial T cells which had passed the positive selection in the thymus through weak binding with the corresponding HLA epitopes [71,72]. Mycobacterial 65 kD heat shock protein (HSP65) shares homology with human HSPs. Clonal expansion of mycobacterial HSP65-reactive T lymphocytes was found in the synovial fluids and blood samples of RA patients. In addition, mycobacterial HSP65 can induce the proliferative response of mononuclear cells derived from RA synovial fluids [73,74]. These studies support the hypothesis that microbial molecular mimicry plays an important role in priming autoimmunity in patients with RA.

### Bystander activation of the immune system

Bystander activation is a process by which microbial products non-specifically activate lymphocytes and immune effecter cells. It has been shown that bystander activation also plays a role in driving the autoimmunity and tissue injury in RA. The pathogen-associated molecular patterns (PAMPs) can bind to the pattern recognition receptors (PRRs) and lead to both innate and adaptive immune cell activation [75]. *P. gingivalis* and *E. coli* LPS induced monocyte activation and the production of RA-associated cytokines interleukin (IL)-1 and IL-33 through the TLR pathways [76,77]. Peptidoglycan, a bacterial cell wall component, is a potent arthritogen. It can activate lymphocytes and induce production of cytokines and polyclonal autoantibodies including RF *in vivo* using animal models and *n vitro* using cell culture systems [78,79].

### Microbial superantigens

Superantigens have long been suggested to play a role in pathogenesis of autoimmune diseases. The frequency of Vβ14^+^ T cells in the synovial fluid of affected joints are significantly higher than that in the peripheral blood of RA patients, implicating that the etiology of RA may involve initial activation of Vβ14^+^ T cells by a Vβ14^+^-specific superantigen [80]. The skewed accumulation of Vβ14^+^ T cells in RA synovial joints was confirmed by another study [81]. EBV infection of human lymphocytes can cause *in vitro* expansion of non-specific B cells and CD8^+^ T cells, leading to polyclonal antibody production and cytotoxic T cell activation [43,82,83]. In animal models, several superantigens, such as mycoplasma arthritidis mitogen and toxic shock syndrome toxin, were able to exacerbate arthritis [50,84].

### Direct effects on joint tissues

Microbial infection can have direct activating or damaging effects on the joint tissues. For example, *Streptococcus pyogenes* infection resulted in the increased expression of receptor activator of NF-κB ligand (RANKL) in mouse osteoblasts in cell culture [85,86]. In another study, Salmonella infection led to RANKL upregulation in synovial fibroblasts derived from mice [87]. Furthermore, co-cultures of Salmonella-infected synovial fibroblasts with osteoclast precursors resulted in the differentiation of multinucleated bone-resorbing, osteoclast-like cells and the formation of bone-resorbing pits [87]. This study provided evidence that Salmonella infection can mediate osteoclast differentiation and activation, which may contribute to bone destruction in infected joints. Recently, it was reported that *P. gingivalis* directly promotes early and later stages of apoptosis of human chondrocytes, which may contribute to the cartilage loss in RA patients [88].

## Conclusion

RA is a complex autoimmune inflammatory disease. The etiopathogenesis of RA involves the interplay of multiple genetic risk factors and environmental triggers. Numerous studies have shown the clinical association of microbial infection with RA. Infection is often detected in early RA and can precede the occurrence of clinical arthritis. These observations suggest that infection contributes to the initiation and exaggeration of RA, arguing against the theory that the RA-associated infection is simply a sequela of immunosuppressive treatments. The pathogenic role of infection in RA is also suggested by studies using arthritis animal models. Among the RA associated microbes, *P. gingivalis* shows the greatest promise as a significant contributor to RA etiology. *P. gingivalis* is the only known prokaryotic organism that contains enzyme peptidylarginine deiminase (PAD) which is essential for the generation of citrullinated autoantigens. Human studies have shown the association of *P. gingivalis* infection with RA patients and individuals at high risk for RA. Animal studies also demonstrated that *P. gingivalis* infection facilitated the development and progression of destructive arthritis. And more interestingly, this effect is dependent on *P. gingivalis* PAD. Future prospective studies examining *P. gingivalis* infection in patients before and at the early-onset of RA using serial collections of patient sera are necessary to confirm the etiopathogenetic role of *P. gingivalis* in RA. Multivariate analyses stratified by RA related factors such as susceptible gene alleles and smoking are also required to pinpoint the role of *P. gingivalis* infection in RA. In addition, studies that elucidate the arthritogenic pathways of *P. gingivalis* infection hold great promise to provide therapeutic targets for the prevention and treatment of RA, a disease affecting 1–2% of the general world wide population.

## Figures and Tables

**Table 1:** Common RA-associated microbes.

Microbes	Clinical association	Animal study	Arthritogenic mechanism
Porphyromonas	Clinical association between RA and periodontitis [6–10]. Presence of *P. gingivalis* DNA in RA patients [17]. Immune responses to *P. gingivalis* in RA patients [34,35]. Increased anti-*P. gingivalis* antibodies in subjects with high risk of RA [36].	Immunization with *P. gingivalis* or *P. gingivalis* enolase induced or exacerbated arthritis [47–49]. *P. gingivalis* facilitated destructive arthritis in CIA mice dependent on its peptidylarginine deiminase [51].	Neo-antigen generation [62]. Molecular mimicry [69]. Bystander activation [47,49]. Direct joint damage [88].
Proteus	Clinical association between RA and urinary tract infection [11]. Immune responses to *P. mirabilis* in RA patients [30–33].		Molecular mimicry [33].
EBV	Clinical association between RA and EBV infection [24]. Presence of EBV DNA and protein in RA patients [21,22]. Immune responses to EBV in RA patients [37,41–43].	EBV induced arthritis in humanized mice [55,56].	Molecular mimicry [70,89]. Superantigen [43,82,83].
Mycoplasma	Presence of DNA [18,19] and glycoglycerophospholipids (GGPL) [29] in RA patients. Immune responses to mycoplasma in RA patients [39,40].	Immunization with mycoplasma arthritidis induced or exacerbated arthritis [46,50,84].	Superantigen [40,50]. Bystander activation [29].
